# Soft palate (dulla) injuries in camels (*Camelus dromedarius*): clinical, hematobiochemical, histopathological findings and treatment outcomes

**DOI:** 10.3389/fvets.2026.1749533

**Published:** 2026-01-30

**Authors:** Madeh Sadan, Faris Aldakhil, Mohie Haridy, Fahad Abdullah Alshanbari, Walid Refaai

**Affiliations:** 1 Department of Clinical Sciences, College of Veterinary Medicine, Qassim University, Buraydah, Saudi Arabia; 2 Department of Surgery, Anesthesiology and Radiology, University Veterinary Hospital, Qassim University, Buraydah, Saudi Arabia; 3 Department of Pathology and Laboratory Diagnosis, College of Veterinary Medicine, Qassim University, Buraydah, Saudi Arabia; 4 Department of Medical Biosciences, College of Veterinary Medicine, Qassim University, Buraydah, Saudi Arabia; 5 Department of Surgery, Anesthesiology and Radiology, Faculty of Veterinary Medicine, Zagazig University, Zagazig, Egypt

**Keywords:** animals, oropharyngeal injury, pathology, pathophysiology, soft palate

## Abstract

The soft palate, particularly the dulla, plays a crucial role in swallowing and maintaining airway function in dromedary camels (*Camelus dromedarius*). Damage to the dulla can negatively impact the health and productivity of these animals.

**Aim:**

This study provides the first detailed description of the clinical, hematobiochemical, and histopathological features of soft palate (dulla) disorders, along with the surgical management of these conditions, in dromedary camels (*Camelus dromedarius*).

**Method:**

A total of 31 dromedary camels diagnosed with dulla disorders were examined through clinical, laboratory, and pathological evaluations.

**Results:**

Seven distinct categories of dulla injuries were identified: lacerated wounds (7; 22.58%), protrusions (7; 22.58%), feed impaction (5; 16.12%), canine tooth puncture wounds (4; 12.9%), abscesses (3; 9.67%), hematomas (3; 9.67%), and gangrene (2; 6.45%). The clinical manifestations varied according to the type and duration of the lesion. Camels with entrapped dulla exhibited anorexia, neck stiffness with extension, and mild respiratory distress, whereas those with dulla protrusion showed swelling, fluctuation, and an inability to retract the dulla into the oral cavity. Analysis revealed A statistically significant increase in levels of sodium (*p* = 0.001), potassium (*p* = 0.008), phosphorus (*p* = 0.026), and ALT (*p* = 0.007), and a significant decrease in calcium (*p* = 0.012), glucose (*p* = 0.001), ALP (*p* = 0.034), and WBC (*p* = 0.03) levels after 2 weeks of treatment, as compared to pre-treatment and control values. The histopathology correlated with clinical findings and injuries varied in severity from erythematous mucosa to gangrene. The lesions included hemorrhages, erythematous mucosa, deep abscesses, lacerations with fresh ulcerations and old ulcers, foreign body pyogranuloma, and gangrene.

**Conclusion:**

A combination of case history, clinical examination, hematobiochemical assessment, and pathological evaluation, together with surgical exteriorization of the dulla, enables an accurate diagnosis of soft palate disorders in dromedary camels. Furthermore, surgical excision of the affected dulla represents an effective and reliable therapeutic approach for managing these conditions in camels.

## Introduction

Dromedary camels (*Camelus dromedarius*) represent a major livestock species in arid and semi-arid regions, contributing significantly to meat, milk production, transportation, and socio-economic stability (1, 2). A camel's overall health plays a crucial role in determining the quantity and quality of its milk and meat production, as well as production efficiency. Disorders of the soft dulla are of particular importance in camels, as they directly affect feeding behavior, hydration status, and overall productivity. Soft palate (dulla) lesions, although considered uncommon, can result in serious functional impairment and welfare concerns, especially in adult males during the rutting season (3, 4). In dromedary camels, the dulla is described as a downward and forward extension of the soft palate in dromedary camels. It is notably more developed in adult males than in female camels and often protrudes outside the mouth cavity during the rutting season, as a display of sexual desire (2, 5–7).

Several types of injuries to the dulla have been reported in adult male camels during the rutting season, including protrusions, lacerated wounds, abscesses, haematomas, impaction feed material, and gangrene (2, 5, 8). These dulla injuries are often caused by the pointed or abrasive edges of the cheek teeth or canines, by she camels during copulation, or by other males during fights (2, 5, 6). Additionally, blunt objects, feed material, and straw can also lead to such injuries. As a result, inflammation accompanied by edematous fluctuating swelling of the dulla may occur, hindering retraction into the mouth cavity and progressively worsening the condition.

Consequently, these injuries are a common reason for performing soft palate surgery in dromedary camels (2, 6). Surgical removal of the injured dulla as a curative procedure has been documented in several previous studies. Additionally, surgical removal of a healthy dulla in adult male racing dromedaries has been employed as a means of enhancing the maximal rate of oxygen consumption during high-speed activity, thereby improving general athletic activity (2, 5).

Although some limited surgical research has been conducted on soft palate injuries in camels, a histopathological investigation of the resected dulla has yet to be performed. Histopathology is important for etiological, pathogenetic, and therapeutic strategies for soft palate injuries. Despite the widespread popularity of camels, as far as the authors are aware, there is only limited detailed research available offering clinical, hematobiochemical, and histopathological findings and means of treatment of soft palate injuries (9). Hence, the present research was carried out to determine clinical, hematobiochemical, and histopathological findings regarding dulla injuries and assess the effectiveness of surgical resection in treating this condition in dromedary camels.

## Materials and methods

### Animals and clinical cases

**Figure 1:**
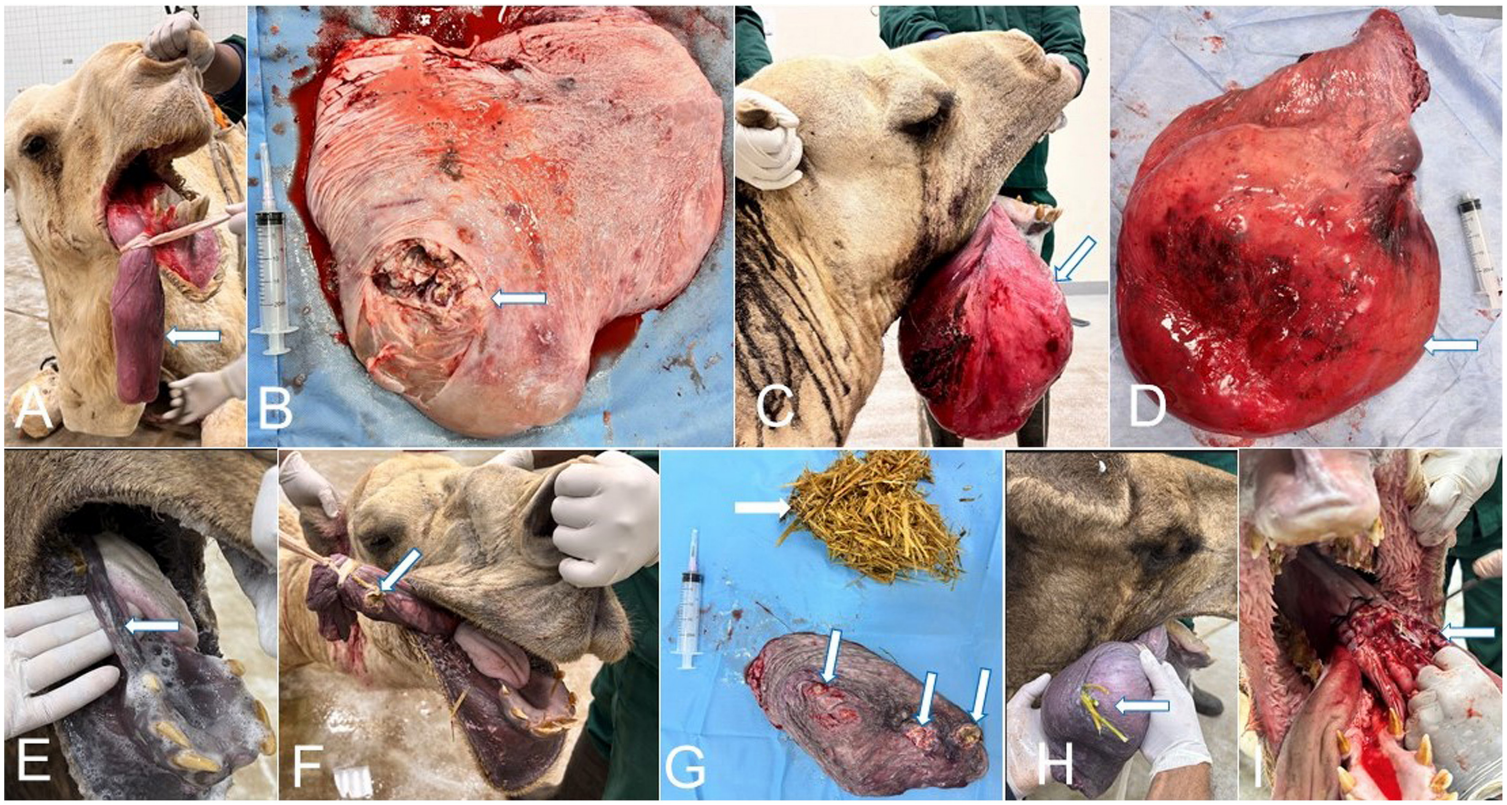
Soft palate lesions and surgical management in camels. **(A)** Abscessated soft palate (arrow). **(B)** Surgical excision of the Abscessated soft palate (arrow). **(C)** Protrusion and hematoma of the soft palate following combat with another male camel (arrow). **(D)** Resected soft palate showing evident hematoma (arrow). **(E)**. Penetrating wound of the soft palate caused by the left canine tooth (arrow). **(F)** Old lacerating wound of the soft palate associated with impacted feed material (arrow). **(G)** Resected soft palate demonstrating multiple lacerations (arrows) and retained feed material (white arrow). **(H)** Soft palate impacted with feed material (arrow). **(I)** Surgical resection of the affected soft palate (arrow).

Thirty-one adult male dromedary camels were presented to the University Veterinary Hospital, Faculty of Veterinary Medicine, Qassim University, Kingdom of Saudi Arabia, between October 2023 and October 2025. Their ages varied between 50 and 130 months (mean ± SD: 89 ± 12 months), with body weights between 470 and 700 kg (mean ± SD: 480 ± 130 kg), and representing various breeds (18 Wadeh, five Asfar, five Ashaal, and three Mejhem). The camels were chosen for inclusion in this research based on their clinical, hematobiochemical, and histopathological characteristics, as well as their medical history of dulla lesions. Various categories of dulla injuries (Figure 1) were documented and classified according to their cause and duration (time from onset of clinical signs to presentation at the clinic; see Table 1). The research protocol was endorsed by the Animal Welfare and Ethics Committee, in compliance with the Laboratory Animal Control Guidelines of Qassim University.

**Table 1:** Clinical findings of soft palate injuries (*n* = 31) in camel.

**Case no**.	^*^Age (years)	**Breed**	**Sex**	**Specific surgical disorder**	**^•^Duration of the injury**
1	8	Wadeh	Male	Abscess	4 days
2	8	Ashaal	Male	Abscess	5 days
3	6	Asfar	Male	Abscess and lacerated wound	5 days
4	8	Mejhem	Male	Entrapment/gangrene	2 days
5	9	Wadeh	Male	Gangrene	5 days
6	5	Wadeh	Male	Hematoma	2 days
7	7	Wadeh	Male	Hematoma	14 h
8	7	Asfar	Male	Hematoma	1 day
9	4	Mejhem	Male	Impaction with feed material	2 days
10	7	Asfar	Male	Impaction with feed material	3 days
11	10	Wadeh	Male	Impaction with feed material	4 days
12	10	Wadeh	Male	Impaction with feed material	2 days
13	5	Wadeh	Male	Impaction with feed material and lacerated wound	2 days
14	6	Wadeh	Male	Lacerated wound	3 days
15	6	Wadeh	Male	Lacerated wound	5 days
16	7	Wadeh	Male	Lacerated wound	5 days
17	8	Ashaal	Male	Lacerated wound	3 days
18	8	Ashaal	Male	Lacerated wound	1 day
19	9	Wadeh	Male	Lacerated wound	3 days
20	11	Ashaal	Male	Lacerated wound	2 days
21	5	Wadeh	Male	Protrusion	2 days
22	6	Mejhem	Male	Protrusion	2 days
23	7	Wadeh	Male	Protrusion	3 days
24	7	Ashaal	Male	Protrusion	1 day
25	8	Asfar	Male	Protrusion	2 days
26	9	Asfar	Male	Protrusion	1 day
27	10	Wadeh	Male	Protrusion	1 day
28	4	Wadeh	Male	Punctured wound by canine	1 day
29	5	Wadeh	Male	Punctured wound by canine	2 days
30	7	Wadeh	Male	Punctured wound by canine	1 day
31	8	Wadeh	Male	Punctured wound by canine	2 days

^*^Age predisposition of dulla lesions was in camels aged 7–8 years, (14 cases, 45.16%). Lacerated wounds (7, 22.58%) and protrusions (7, 22.58%) were the prevalent lesions.

^•^The duration of injury was defined as the time elapsed from its occurrence until presentation to the clinic.

### Clinical examination

Clinical examinations were performed on the subject camels with dulla injuries to assess the physical features of the injured dulla, including the Etiology, category, and duration of the injury. Each camel's age, breed, and gender were also documented. All of these criteria were assessed and correlated among the examined animals. The types of soft palate lesions observed across various camel breeds (*n* = 31) are summarized in Table 1.

### Hematological and biochemical examination

Hematological and biochemical examinations were carried out for all included camels, both pre-operative and 2 weeks after surgical intervention. For the Hematological examination, a 5 ml blood sample was collected via jugular venipuncture from each camel and deposited into EDTA-containing tubes. Samples were analyzed using an automated hematology analyzer (VetScan HM5, Abaxis, Union City, CA, USA). A CBC test was performed to evaluate blood parameters, including total and differential leukocyte counts, red blood cell count (RBC), hematocrit (HCT), hemoglobin concentration (HGB), mean corpuscular volume (MCV), mean corpuscular hemoglobin (MCH), and mean corpuscular hemoglobin concentration (MCHC). For the hematobiochemical examination, a 10 ml blood sample was collected from each camel via jugular venipuncture and collected into plain tubes without anticoagulant. The samples underwent centrifugation (Hermle Z 306, Labortechnik, Germany) at 4,500 rpm for 20 min to separate the clear serum. Serum biochemical parameters were analyzed using an automated analyzer (VetScan VS2, Abaxis, Union City, CA, USA), including total protein (TP), albumin (ALB), globulin (GLOB), glucose (GLU), creatinine (CR), creatine kinase (CK), blood urea nitrogen (BUN); liver enzymes; aspartate aminotransferase (AST), alanine aminotransferase (ALT), γ-glutamyl transferase (GGT), alkaline phosphatase (ALP); and electrolytes and minerals calcium (Ca), phosphorus (P), magnesium (Mg), sodium (Na), potassium (K). The obtained biochemical values were compared with reference data previously reported by (3, 10).

### Surgical technique

Surgical amputation of the affected dulla was performed as a curative approach to alleviate clinical manifestations, following thorough clinical and hematobiochemical assessments. For management of infection, control of inflammation, pain, and stabilization of vital functions; camels scheduled for surgery received intravenous fluid therapy consisting of 0.9% normal saline solution (1–3 L; Saudi Pharmaceutical Solution Industry, Riyadh, Saudi Arabia) and a 5% dextrose infusion (1–2 L; Saudi Pharmaceutical Solution Industry, Riyadh, KSA). Prior to surgery, penicillin (30,000 IU/kg) and streptomycin (10 mg/kg) were administered intramuscularly (Pen & Strep, manufactured by Norbrook Laboratories, UK), along with flunixin meglumine (1.1 mg/kg; Finadyin, Schering-Plough, UK). Sedation of the diseased camels was achieved through an intravenous administration of 0.3 mg/kg xylazine HCl.

Each affected camel was restrained and secured in a sternal recumbent position, and the entrapped injured dulla gently exteriorized using long sponge forceps, while light digital pressure was applied over the enlarged pharyngeal region. The protruding injured dulla was grasped with a sterile surgical towel and extended as much as possible to allow for aseptic preparation. A local infiltration anesthesia of 2% lidocaine hydrochloride (Norbrook Laboratories, UK) was administered at the base of the dulla, following standard aseptic preparation of the surgical site. After achieving an adequate level of local anesthesia, interrupted overlapping horizontal mattress sutures were placed to achieve hemostasis, using absorbable suture material (coated polyglactin 910, No. 2; manufactured by United Medical Industries Co., Ltd., Riyadh). The injured dulla was amputated 3 cm distal to the suturing line, and the wound sutured in a routine manner with a simple continuous suture pattern.

### Postoperative management and follow-up

Postoperatively, the antibiotic and anti-inflammatory regimens initiated before surgery were maintained for five consecutive days, accompanied by an intramuscular administration of 10 ml of vitamin AD3E solution (ADVIT-DE, produced by Morvel Laboratories P. Ltd.). After operation, the camels were kept under stall confinement for 1 month, during which daily clinical observation was performed to monitor wound healing and overall recovery. The animals were discharged approximately 4 weeks after surgery, following satisfactory healing. To assess the long-term clinical outcomes, a telephone follow-up was conducted 6 months after the operation. Owners were interviewed regarding the presence of any postoperative discharge and the functional outcome of the procedure.

### Statistical analysis

The data were handled and analyzed using a commercial software program (Graph Pad prism ver. 9.2, USA). The Kolmogorov–Smirnov test was used to evaluate data distribution, and the results confirmed normal distribution. Thus, the results were presented as mean and standard deviation. To assess the effect of treatment on hematological and biochemical parameters, an unpaired *t*-test was used. The results of post-treatment were compared to those of pre-treatment and control. When the *p*-value was < 0.05, the results were considered significant.

### Histopathological examination

The injured dulla was surgically resected and tissue samples fixed in 10% neutral buffered formalin. For histopathological evaluation, two 5-μm sections were prepared and stained using hematoxylin and eosin (H&E). Slide assessment was performed independently and in a blind manner by a board-certified veterinary pathologist.

## Results

### Clinical findings

Seven categories of dulla injuries were reported in this study. Lacerated wounds (7, 22.58%) and protrusions (7, 22.58%) were the prevalent lesions, followed by impactions of the dulla with feed (5, 16.12%), puncture wounds by canines (4, 12.9%), abscesses (3, 9.67%), hematomas (3, 9.67%), and gangrene (2, 6.45%) as less frequent lesions (see Table 1). Camels with entrapped dulla showed dysphagia, neck stiffness, and mild respiratory distress, accompanied by ulcerations, feed impactions, hematomas, gangrene, fibrosis, and penetrating wounds caused by camel canines or other foreign objects. In contrast, protruding dulla appeared swollen and fluctuating, with the affected camels unable to retract them into their mouths.

In the present study, lacerations and puncture wounds (11, 35.48%) were caused by long and sharp teeth, especially canines, and impacted feed material. Three cases (9.67%) developed hematomas due to sharp injuries inflicted by canines and molars, resulting in submucosal hematoma formation. Gangrene was the least frequent lesion (2, 6.45%), following lacerated wounds and hematomas. Impaction of the soft palate by feed material was observed in five camels (16.12%), presenting as a heavy mass difficult to exteriorize from the throat region; flushing with warm water facilitated management in these cases. Dulla protrusion was noted in seven camels (22.58%), occurring as a result of feed impaction, pursuit of females for mating, or aggressive interactions with other males. Successful treatment of all dromedary camels enrolled in this research was accomplished through surgical amputation of the injured soft palate (see Figures 1A–I).

Regarding breed susceptibility, dulla lesions were more prevalent in the Wadeh breed (18 cases, 58.06%) than Asfar (5, *p* = 0.0013), Ashaal (5, *p* = 0.0013), and Mejhem (3, *p* < 0.001). However, there were no significant variations among the other breeds. The age predisposition of dulla lesion was evidenced in animals at 7–8 years of age. Fourteen camels (45.16%) were found affected at this age. However, old camels (≥ 11 years, 1 case, 3.22%) were less affected when compared with camels at 7–8 years (*p* < 0.01). Single dulla lesion was significantly higher than mixed lesion (29 vs. 2, *p* < 0.001). Lacerated wounds (7, 22.58%) and protrusions (7, 22.56%) were prevalent. Abscesses (3, 9.67%) and gangrene (2, 6.45%) were less frequent. Impaction from feed (5, 16.12%), puncture wounds from canines (4, 12.9%), and hematomas (3, 9.67%) were also present. Regarding owner observation of dulla lesion, prompt response to the signs and rapid examination of the camel occurred within 14 h of lesion (1, 3.22%). Delayed response and veterinarian consultation were recorded 5 days after onset (3 cases, 9.67%).

### Hematological and biochemical findings

**Figure 2:**
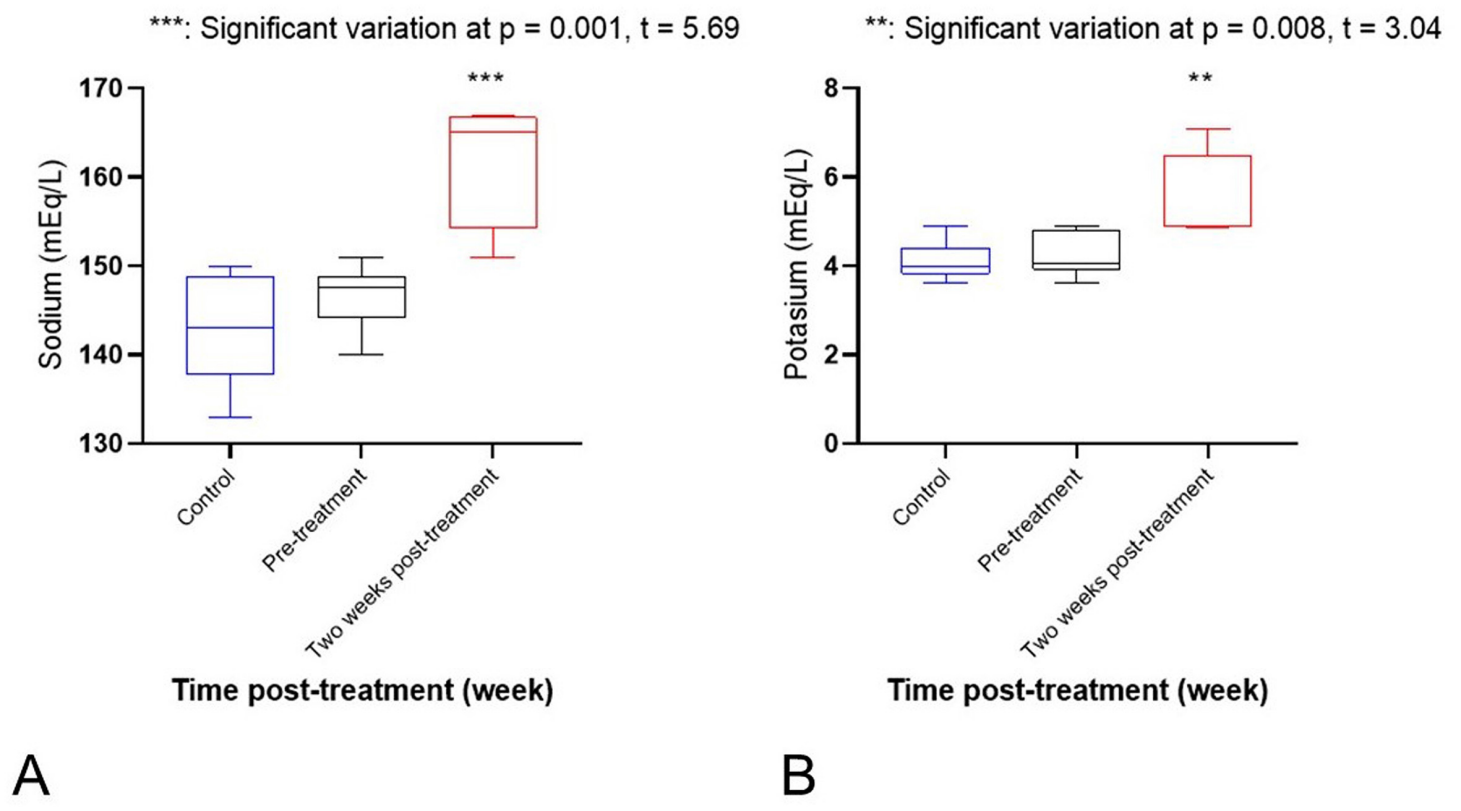
**(A)** A significant increase of sodium (*p* = 0.001). ***Significant variation at *p* = 0.001, *t* = 5.69. **(B)** A significant increase of potassium (*p* = 0.008) level after 2 weeks of treatment in comparison with pre-treatment and control values. **Significant variation at *p* = 0.008, *t* = 3.04.

**Figure 3:**
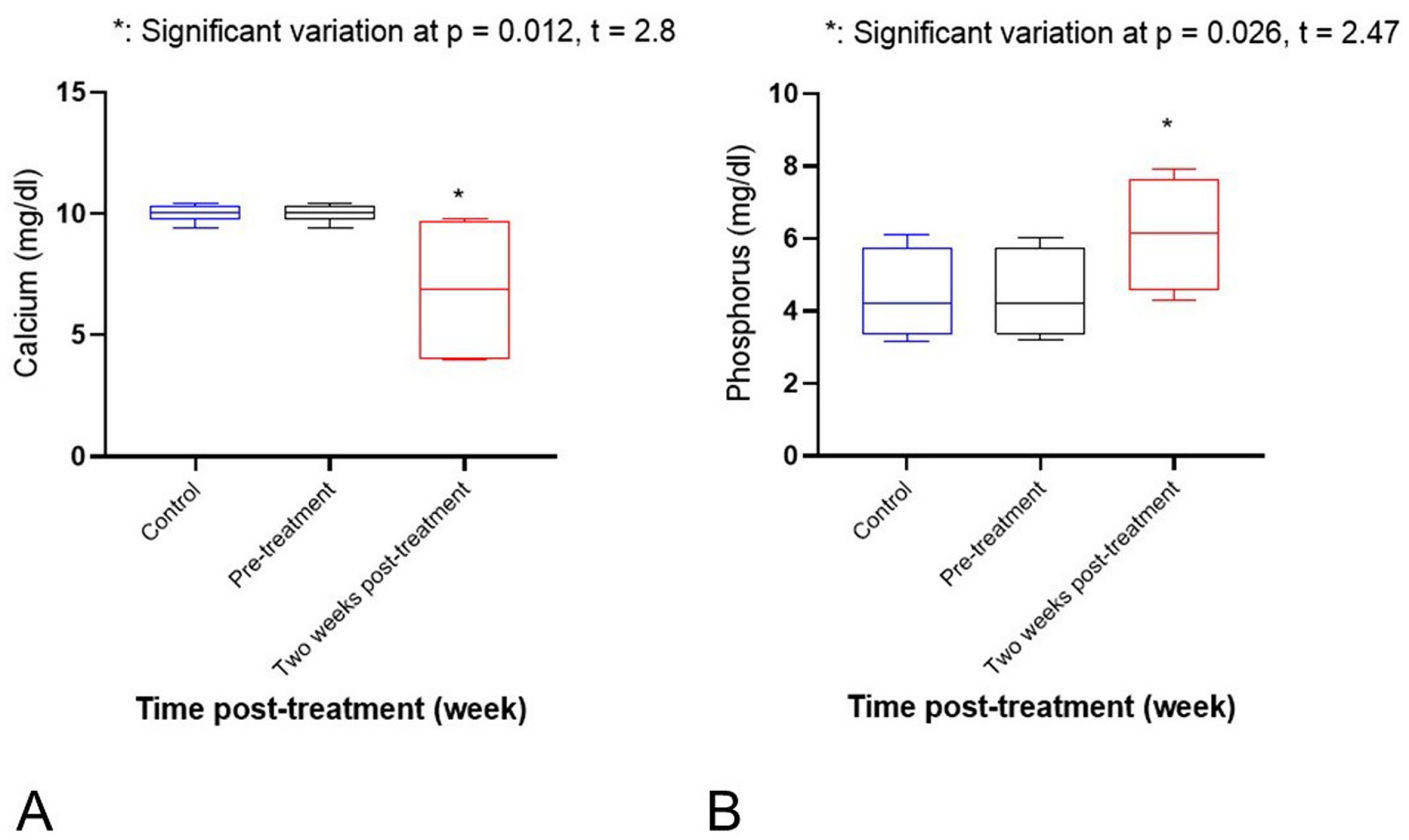
**(A)** A significant decrease of calcium (*p* = 0.012). *Significant variation at *p* = 0.012, *t* = 2.8. **(B)** A significant increase of phosphorus (*p* = 0.026) level after 2 weeks of treatment in comparison with pre-treatment and control values. *Significant variation at *p* = 0.026, *t* = 2.47.

**Figure 4:**
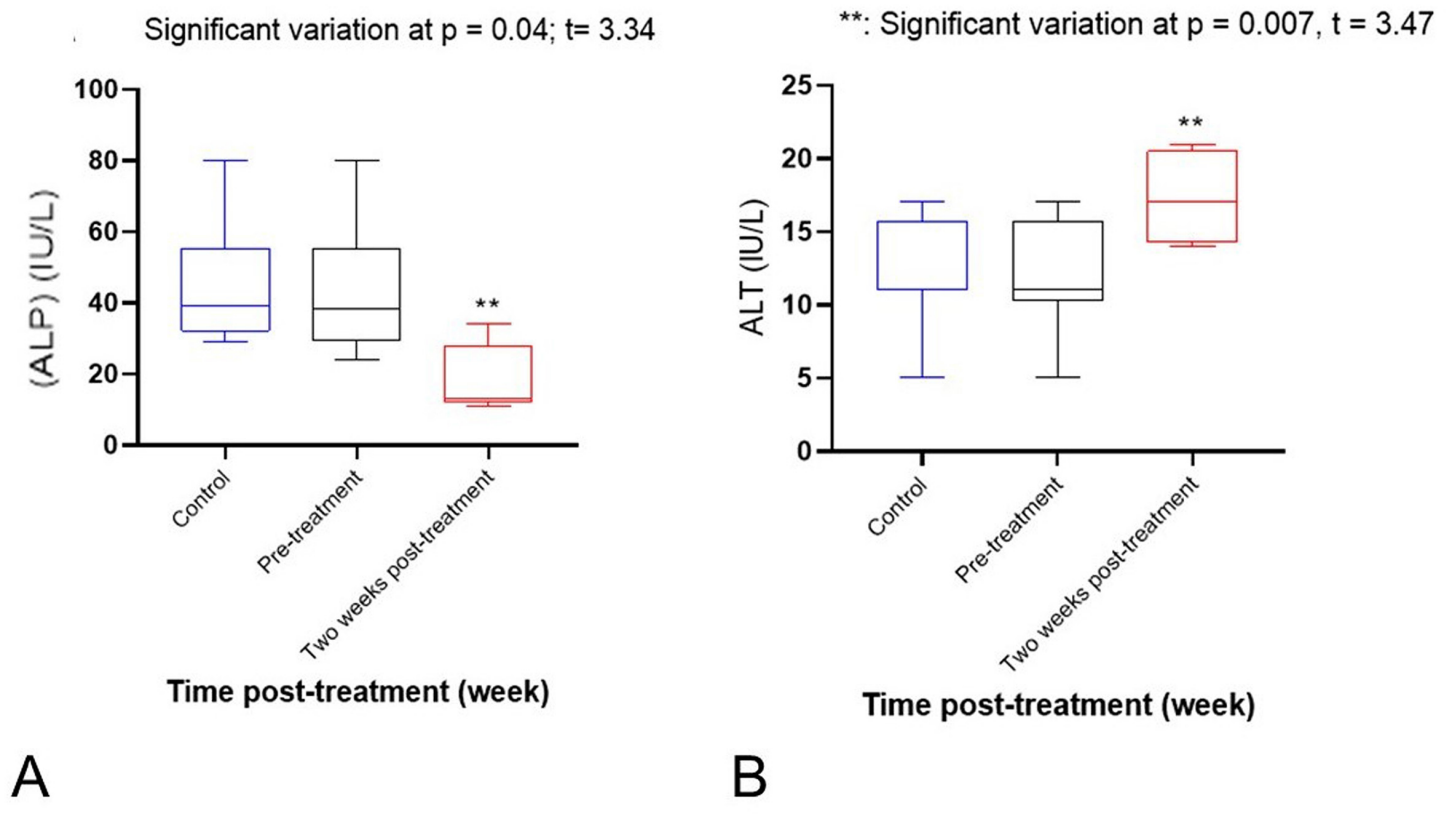
**(A)** A significant decrease of serum alkaline phosphatase (ALP; *p* = 0.034). *Significant variation at *p* = 0.04, *t* = 3.34. **(B)** A mild increase of alanine aminotransferase (ALT; *p* = 0.007) level after 2 weeks of treatment in comparison with pre-treatment and control values. **Significant variation at *p* = 0.007, *t* = 3.47.

**Figure 5:**
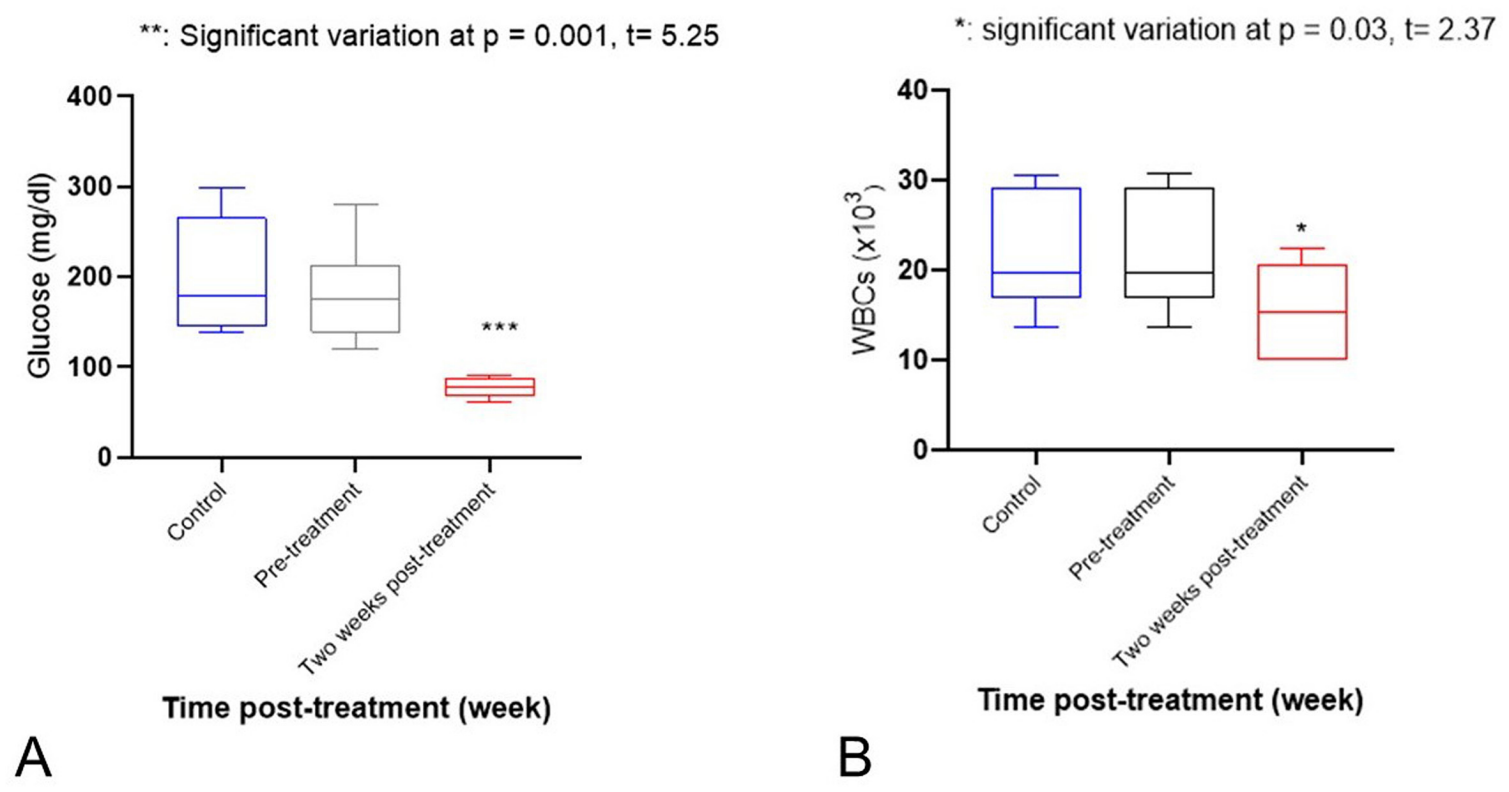
**(A)** A significant decrease of Serum glucose (GLU; *p* = 0.001). **Significant variation at *p* = 0.001, *t* = 5.25. **(B)** significant decrease of White blood cells (WBCs; *p* = 0.03) level after 2 weeks of treatment in comparison with pre-treatment and control values. *Significant variation at *p* = 0.03, *t* = 2.37.

Regarding electrolytes, there was a significant increase in sodium (*p* = 0.001) and potassium (*p* = 0.008) levels after 2 weeks of treatment, as compared to pre-treatment and control values (see Figures 2A, B). Serum calcium showed a significant decrease after 2 weeks of treatment, as compared to pre-treatment and control values (*p* = 0.012). However, phosphorus showed a significant increase (*p* = 0.026; see Figures 3A, B). Activity of serum alkaline phosphatase (ALP) was significantly decreased after 2 weeks of treatment, as compared to pre-treatment and control values (*p* = 0.034). However, alanine aminotransferase (ALT) showed a mild increase (*p* = 0.007; see Figures 4A, B). There were non-significant changes in aspartate aminotransferase (AST; *p* = 0.5), gamma-glutamyl transferase (GGT; *p* = 0.2), and creatine kinase (CK; *p* = 0.3). No significant variations were observed in TP, ALB, GLOB, BUN, CR, or AMY levels. Serum glucose (GLU) showed a significant decrease after 2 weeks of treatment, as compared to pre-treatment and control values (*p* = 0.001; see Figure 5A). Regarding hematological variables, RBCs count, hemoglobin, and PCV% showed non-significant changes between groups. Also, other hematological indices (MCH, MCHC, RDWc, and RDWs) showed non-significant changes. White blood cells (WBCs) showed a significant decrease after 2 weeks of treatment, as compared to pre-treatment and control values (*p* = 0.03; see Figure 5B). However, other differential Leukocyte counts showed non-significant variations.

### Histopathological findings

**Figure 6:**
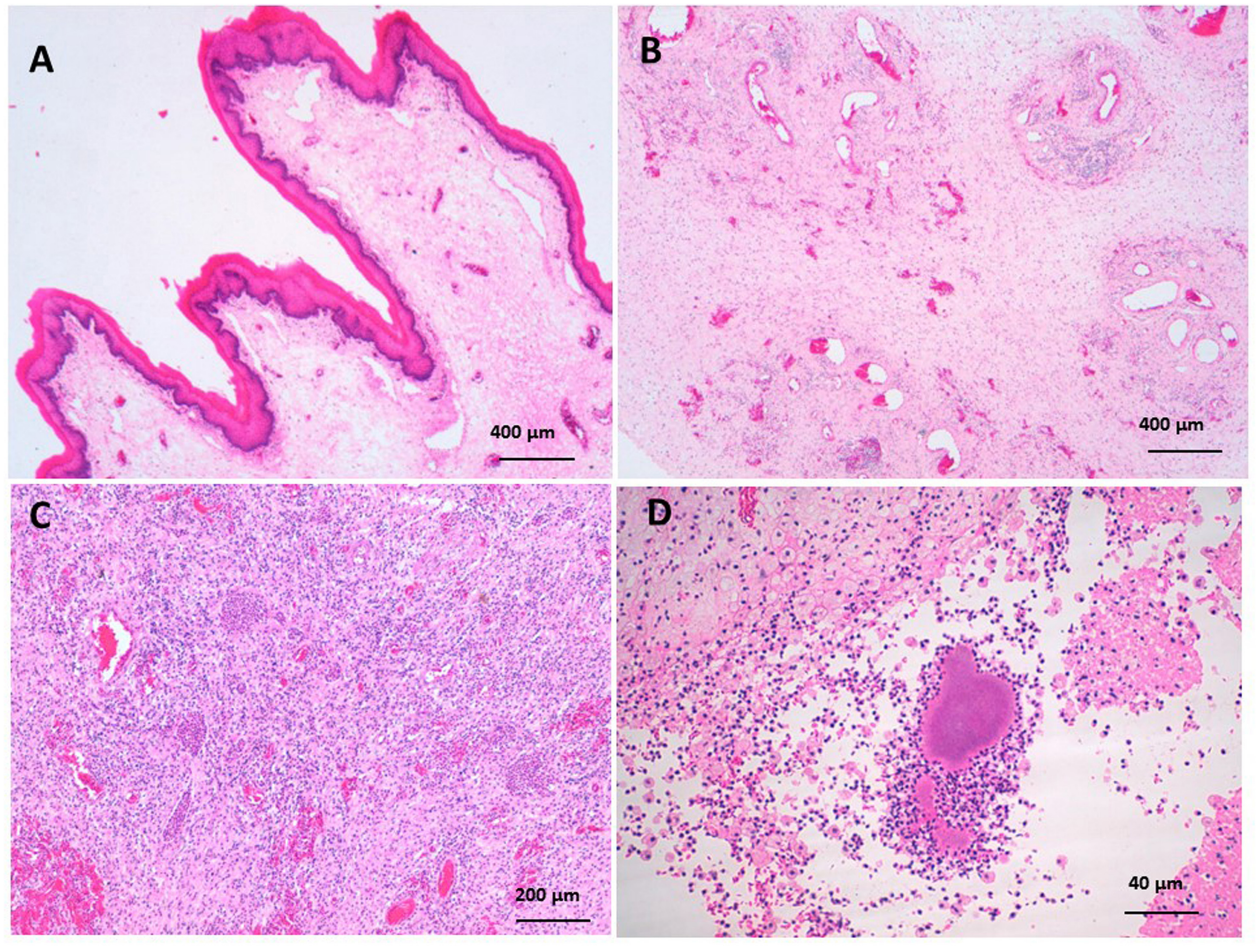
**(A)** The soft palate is lined with a mucosal layer consisting of stratified squamous epithelium. The lamina propria contains connective tissue, lymphatic tissue. **(B)** Severe suppurative vasculitis characterized with intravascular and perivascular neutrophilic infiltration in the submucosal vasculature. **(C)** abscesses and diffuse suppurative inflammation associated with venal thrombosis with pyogenic emboli. **(D)** Colonies of actinomyces were observed related to abscesses.

**Figure 7:**
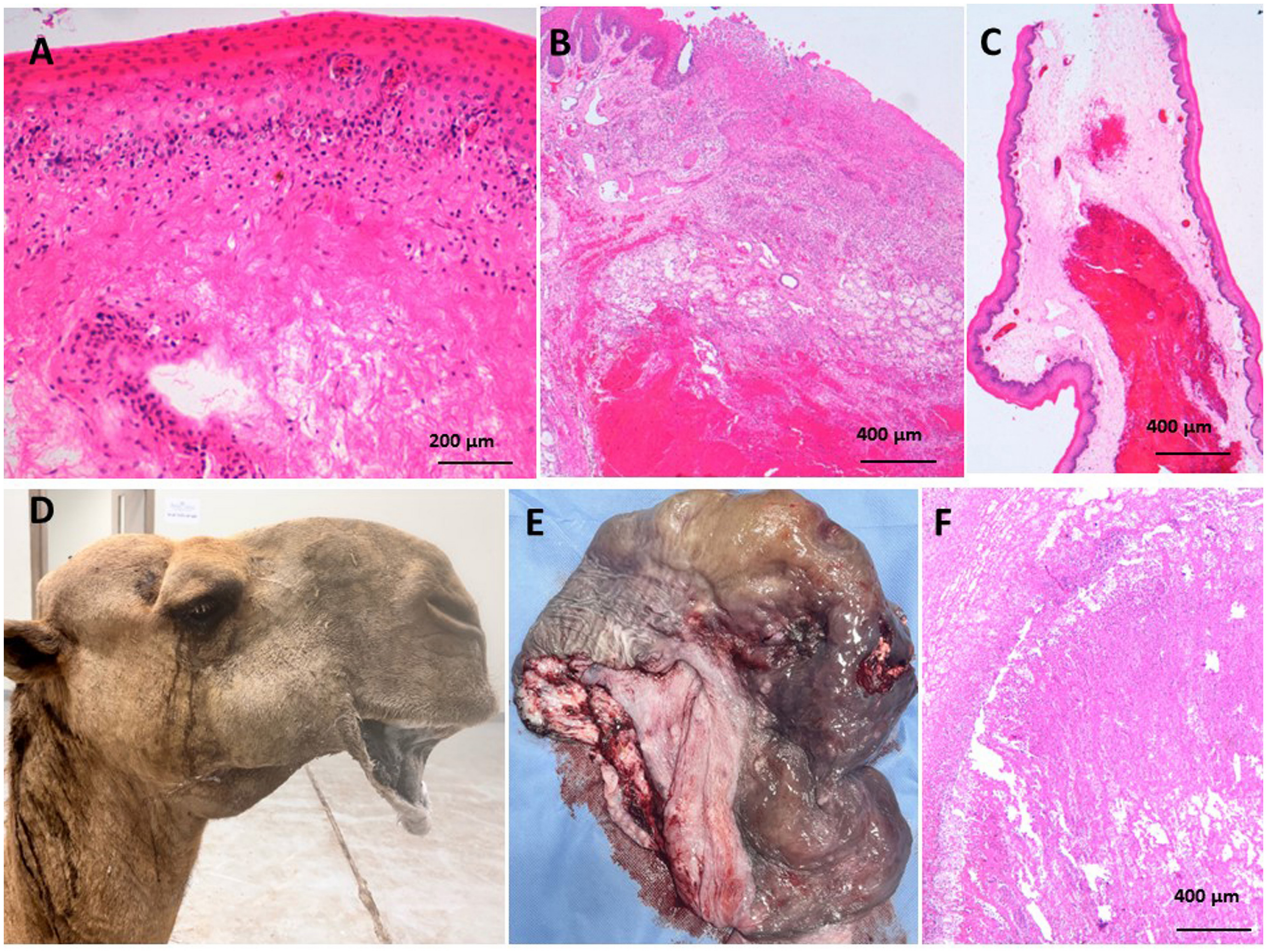
**(A)** Erythematous mucosa characterized by mucosa was infiltrated singly with neutrophils however the mucosa-submucosa junctions were densely infiltrated with neutrophils as well as around submucosal blood vessels. **(B)** Hemorrhages and abscesses were observed in the submucosa and overlying mucosa were ulcerated and healed with granulation tissue. **(C)** Areas of massive hemorrhages were observed in submucosa. **(D)** Severe pain due to impaction (entrapment) of soft palate between teeth and check. Lacrimation and drooping of lips and immobilization of head and neck are clinical signs. **(E)** Gangrenous necrosis of soft palate revealed brackish discoloration, swelling and gas bubble formation. **(F)** Large gangrenous area revealed necrotic center and surrounding alive suppurative inflammation.

The pathological findings of the resected dullas varied from simple erythematous lesions to severe gangrenous necrosis. The pathological lesions corresponded to the clinical findings of the affected camels. Normally, the dulla is rose pink with a wet surface, movable, and can be protruded and returned easily to the oral cavity, Histologically, the soft palate is a muscular sheet lined with mucosal layers consisting of stratified squamous epithelium in the oro- and laryngopharynx on the ventral surface, and pseudostratified ciliated columnar epithelium in the nasopharynx on the upper side. The lamina propria contains connective tissue, lymphatic tissue, and glands, with regional variation in density (see Figure 6A). The abscessed dulla shown in Figures 1A, B reveal severe suppurative vasculitis in the submucosa, characterized by intravascular and perivascular neutrophilic infiltration in the submucosal vasculature (see Figure 6B). The pyogenic vasculitis progressed to thrombosis and liquefactive necrosis, leaving abscesses and diffuse suppurative inflammation. Venous thrombosis with pyogenic emboli was also observed. Colonies of actinomyces were found, related to abscesses in the vasculature (see Figures 6C, D). In the soft palate hematomas (see Figures 1C, D), the histopathology revealed erythematous mucosa characterized by heavy infiltration of neutrophils at the mucosa-submucosa junction and around submucosa blood vessels (see Figure 7A). The submucosa was edematous.

Punctures (see Figure 1E) and lacerations of the soft palate (see Figures 1F, G) due to impaction of food materials revealed hemorrhages and abscesses in the submucosa and overlying mucosa, which ulcerated and healed with granulation tissue (see Figure 7B). In hemorrhagic soft palates, massive hemorrhages were observed in the submucosa (see Figure 7C). Edema of the areolar connective tissue featured diffuse neutrophil cell infiltration, especially around blood vessels. In impacted soft palates in between the cheek and masseter muscles, severe gangrenous lesions were observed. Clinically, severe pain due to impaction of the soft palate between teeth and check, lacrimation and drooping of lips, and immobilization of the head and neck was observed (see Figure 7D). Gangrenous necrosis of the soft palate revealed blackish discoloration, swelling, and gas bubble formation (see Figure 7E). Large gangrenous areas revealed necrotic centers and surrounding live suppurative inflammation. The necrotic areas were rich with saprophytic bacteria and mineral precipitation (see Figure 7F). Old healed contracted ulcers appeared star-like, leaving deep ulcerated centers and raised fibrosed edges. Microscopically, the mucosa was thick, wrinkled, and corrugated and the overlying epithelium was hyperplastic. The submucosa was infiltrated with remnants of chronic inflammatory cells, especially around blood vessels.

### Treatment outcomes

A clear relationship was observed between the duration of dulla injury and overall health status of the affected dromedary camels, along with the condition of the entrapped or protruded dulla. Early diagnosis, timely intervention, and appropriate post-operative care were associated with favorable treatment outcomes. Post-operative follow-ups were conducted over a 6-month period through telephone contact and field visits, focusing on the effects of dulla amputation on eating and swallowing ability before and after surgery, the presence of any abnormal discharge from the surgical site, and final functional outcome. All of the treated camels achieved complete recovery by 6 months post-surgery.

## Discussion

Injury to the soft palate (dulla) is a prevalent and severe condition in dromedary camels that frequently requires surgical intervention to address complications (2, 6). Because of limited knowledge offered in the available literature about clinical, histopathological, and hematobiochemical findings related to dulla injuries in dromedary camels and their prevalence, the present study examined dulla disorders and assessed the outcomes of surgical excision with regards to managing such conditions. The prevalence of soft palate lesions across different camel breeds was also assessed. Among the studied breeds, Wadeh camels exhibited the highest prevalence (18 cases, 58.06%), as compared to other breeds (13 cases, 41.94%). This higher prevalence could be due to the relatively larger population of Wadeh camels in Saudi Arabia, reflecting their greater productive, reproductive, and economic significance (1, 11, 12).

Clinical examination is routinely used to diagnose dulla disorders in dromedary camels; however, surgical exteriorization, along with hematobiochemical and histopathological assessments, can provide definitive confirmation, especially when history and clinical signs are inconclusive. In this study, the clinical, hematobiochemical, and histopathological features of dulla injuries varied with their type and duration, these findings are consistent with (2, 5).

The dulla constitutes the ventrally oriented diverticulum of a camel's soft palate, forming a sac-like structure that can inflate during vocalization or social interactions. It plays a role in thermoregulation, sound production, and in males, may be more pronounced due to sexual dimorphism (13). In the present study, seven distinct categories of dulla injuries were systematically identified and accurately diagnosed, including lacerated wounds (7; 22.58%) and protrusion (7; 22.58 %), which were the most prevalent lesions, followed by impaction of the dulla from feed (5; 16.12 %), puncture wounds by canines (4; 12.9 %), abscesses (3; 9.67%), hematomas (3; 9.67 %), and gangrene (2; 6.45 %), which were less frequent. In the studied camels, entrapped dulla manifested as dysphagia, mild dyspnea, and swelling in the neck region, whereas protruded dulla appeared as a large, edematous mass protruding from the oral cavity, with the camel unable to retract it into the oral cavity. Comparable findings have been reported by a number of other researchers (2, 5, 6, 8). Dulla injury represents a common surgical condition in dromedary camels, predominantly resulting from sharp trauma caused by elongated canines and molars, impacted feed, or tree branches, as well as from male-to-male aggression and the pursuit of females during the rutting season. In the present study, all affected camels underwent successful surgical resection. These findings are consistent with other research on this topic (2, 9). Our findings demonstrate that male camels exhibited a 100% prevalence (*n* = 31) of dulla injuries, which may be due to the more prominent dulla structure in males as compared to females. Similar observations were reported by (9).

In this study, clinical cases of dulla injury in camels were identified and compared to healthy controls according to (3, 10). Diagnosis was established based on clinical, hematological, biochemical, and histopathological evaluations. Our findings demonstrated a significant reduction in serum calcium and glucose concentrations in camels affected with dulla injuries. These alterations may reflect prolonged dysphagia, reduced feed intake, and dehydration prior to presentation, which could persist transiently during the early post-treatment period. Hypocalcaemia in particular may be exacerbated by impaired dietary intake and altered metabolic demands associated with stress and inflammation. In contrast, the significant elevation in serum phosphorus may be explained by the well-recognized inverse relationship between calcium and phosphorus homeostasis, whereby reduced calcium availability is accompanied by a compensatory rise in phosphorus concentrations. Similar biochemical patterns have been reported in previous studies investigating metabolic and nutritional disturbances in camels with oropharyngeal and soft tissue injuries (3, 10, 14–16). The significant increase in serum sodium and potassium levels observed 2 weeks after treatment, compared with both pretreatment and control values, may be associated with dehydration, anorexia, and inflammatory responses related to dulla injury. Fluid imbalance and reduced water intake can lead to hemoconcentration and electrolyte derangements, while tissue injury and inflammation may further influence transcellular electrolyte shifts. These interpretations are consistent with earlier reports describing electrolyte disturbances in diseased camels (16). Regarding enzyme activity, the significant decrease in serum alkaline phosphatase (ALP) alongside a mild increase in alanine aminotransferase (ALT) may indicate a systemic response to inflammation, stress, and reduced nutritional intake following acute dulla injury. Although these enzymatic changes are not specific, they may reflect altered metabolic activity rather than primary hepatic pathology. Comparable trends in ALP and ALT activities have been documented in camels suffering from inflammatory or traumatic conditions affecting the oral and pharyngeal regions (10, 15, 17). Hematological parameters in camels with dulla disorders were largely comparable to those of the controls. However, a significant reduction in white blood cell counts (leukopenia) was recorded 2 weeks after treatment compared with pretreatment and control values. This finding may be related to stress-induced immunosuppression, possibly mediated by elevated cortisol levels following injury and handling, rather than an ongoing infectious process. Similar hematological responses have been described in previous studies evaluating stress and inflammatory conditions in camels and other large animals (3, 10, 15, 17). All 31 surgically treated camels achieved complete recovery with full restoration of normal performance. Similar results were obtained by other researchers (2, 9).

The soft palate of the camel consists of a core of skeletal muscle fibers with a mucosa covering, ventrally by a non-keratinized stratified squamous epithelium and dorsally with pseudostratified squamous epithelium (18). Gross pathological lesions such as lacerations, feed impaction, protrusions, canine-inflicted puncture wounds, gangrene, and hematoma of the soft palate have previously been documented in camels (2, 5, 19). Histopathology of the resected dulla has not been performed. Histopathology requires deep knowledge of pathogenesis for surgical problems. In the present study, the lesions varied in severity from erythematous lesions to severe gangrene. Erythematous lesions of the muco-submucosal junction are related to allergic conditions; especially, inflammation is localized around blood vessels (20). Hemorrhages, punctures, and lacerations from sharp teeth have previously been observed. Deep thorn associated with Sulfur granules, abscesses, and histologically correlated actinomyces colonies have previously been observed in lamas with mandibular infections and tooth root abscesses (21). Actinomyces were isolated as normal flora from the nose, paranasal sinuses, and oral cavity (22). Moreover, ulcerated wounds with granulation tissue formation, as well as old star-like healed ulcers, were also observed. Gangrene of entrapped or impacted parts of the soft palate between the cheek muscles and mandible were also observed. Gangrene might occur due to venous congestion resulting from vascular compression leading to tissue necrosis and following saprophytic invaders that turn to gangrene (2, 5, 19).

## Conclusion

Accurate diagnosis and informed surgical management of various dulla injuries in dromedary camels can be reliably achieved through a comprehensive diagnostic approach that integrates detailed case history, thorough clinical examination, and careful surgical exteriorization of the affected dulla. The inclusion of hematobiochemical analyses contributes valuable insight into the systemic health status of affected animals, while histopathological evaluation provides definitive confirmation of lesion type and severity. Collectively, these diagnostic tools enhance clinical decision-making and allow for appropriate therapeutic planning. Surgical excision of the injured dulla was demonstrated to be a safe and effective curative intervention, yielding favorable clinical outcomes and satisfactory postoperative recovery. Early diagnosis and timely surgical management remain critical to reducing complications, improving animal welfare, and restoring normal physiological function. Despite these encouraging findings, significant research gaps remain. Available data on dulla-related lesions in dromedary camels are largely limited to case reports and small case series, with a lack of large-scale, multicenter, or longitudinal studies assessing incidence, risk factors, and long-term outcomes. Standardized diagnostic criteria, surgical protocols, and postoperative management guidelines are not yet well-established. Furthermore, the pathogenesis, progression, and potential breed-, age-, or management-related predispositions to dulla injuries remain poorly understood. The role of advanced imaging modalities, molecular diagnostics, and minimally invasive surgical techniques has also not been adequately explored in this species.

## Data Availability

The datasets presented in this study can be found in online repositories. The names of the repository/repositories and accession number(s) can be found in the article/supplementary material.
